# Clues to Our Past: Mining the Human Genome for Signs of Recent Selection

**DOI:** 10.1371/journal.pbio.0040094

**Published:** 2006-03-07

**Authors:** Liza Gross

Within the past 100,000 years, *Homo sapiens* left Africa in search of new opportunities, likely crossing paths with *H. erectus* in Asia and *H. neanderthalensis* in Europe. These early pioneers encountered unfamiliar climates, habitats, and food sources (not to mention alien human species). Then, after adjusting to a major climate change following the last ice age, they underwent a dramatic lifestyle switch, from hunting and gathering to agriculture—a change that brought crowded living conditions and new infections. All these radical changes likely precipitated significant genetic adaptations, with selection favoring genotypes most suited to the novel conditions. Indeed, recent studies have found evidence of strong selection on new gene variants reflecting adaptations to disease (conferring resistance to malaria) and dietary changes (lactose tolerance).

In a new study, Benjamin Voight, Sridhar Kudaravalli, Jonathan Pritchard, and their colleagues take a global approach to search for such signals across the genome and characterize the types of gene variants, or alleles, targeted by selection. Analyzing variants called single nucleotide polymorphisms (SNPs) in three populations—one sampled from Africa; one, a combined sample of Japanese and Chinese individuals from Asia; and one from Europe—they found widespread signals of recent positive selection in all three populations. These signals highlight the types of biological processes that have been targeted by selection during the evolution of modern humans.

Voight et al. based their analysis on data recently collected by the HapMap Project, a repository of the SNPs that researchers use to identify genetic variants involved in human health and disease risk. Humans differ by just 0.1% at the nucleotide level, but these polymorphisms and the linked loci they are inherited with (called haplotypes) contain a wealth of clues to our evolutionary history.

The authors analyzed about 800,000 SNPs from 309 individuals, looking for genomic regions where strong selection has pushed new alleles to intermediate frequency. While these polymorphisms could become fixed (widespread in the population) or remain as SNPs, they likely reflect recent adaptations. To systematically detect this type of signal across the genome, they developed a new statistical scoring method (called integrated haplotype score, or iHS) that builds on an existing test for positive selection.

To detect selection, both tests rely on the relationship between an allele's frequency and the distance it maintains with homozygous, or invariable, loci that occur on either side of it along the chromosome (referred to as extended haplotype homozygosity). Old alleles with high population frequency will have only short-range correlations with adjacent loci; variants that quickly increase in frequency due to selection tend to sit within longer haplotypes with lower nucleotide diversity than predicted under models of neutral evolution. By scoring the strength of selection on an SNP, the new test identifies SNPs that stand out against the rest of the genome. Since strong selection is likely to sweep large blocks of adjacent loci along the path to fixation, the authors focused on SNP clusters with extreme iHS scores rather than on individual SNPs.

The authors saw extreme values clustered within distinct genomic regions, including a region containing the lactase gene and other known targets of strong selection. Additionally, when they looked for the strongest signs of selection, they found many regions classified as selection candidates by the HapMap Project in their results. Most of the detected signals occurred recently, based on the lengths of the haplotypes. And since most of the selection signals are found in different populations, they likely occurred after the populations segregated, during the agricultural transition. Many of the selected genes play a role in fertility and reproduction (relating to sperm motility and egg fertilization) and morphology (relating to skin pigmentation and skeletal development). Others are involved in food metabolism, reflecting regional changes in diet.

With this genome-wide map of selected targets, future studies can explore what types of selective pressures these genes likely responded to and fill in the gaps in our recent evolutionary history. Although the nature and target of these selection signals are not yet clear, the authors argue that many might have medical implications and that such “selection maps” should become an integral part of annotating the human genome sequence. To facilitate this application, they have created files of SNPs that can be used to tag the strongest selection signals and a Web tool (http://pritch.bsd.uchicago.edu/data.html) for querying any HapMap SNP for evidence of selection. And by providing a more sensitive method of detecting selection, the authors' new test statistic should prove a valuable tool for uncovering the significant events in our history that lay hidden in the sequence of our DNA.

## 

**Figure:**
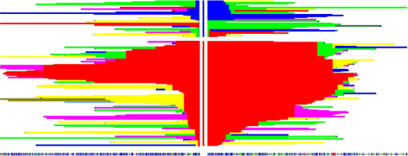
In this graphical representation, a new selected allele (red, center) is sweeping to fixation, replacing the ancestral allele (blue).

